# The effects of mentorship and educational videos on nursing students’ physical examination skills: a clinical audit

**DOI:** 10.1186/s12912-023-01626-w

**Published:** 2023-12-06

**Authors:** Mohammad-Amin Nasiri, Mahdieh Sabery, Mahboubeh Rezaei, Hamidreza gilasi

**Affiliations:** 1 Medical Surgical Nursing Department, School of Nursing and Midwifery, Kashan University of Medical Sciences, Kashan, Iran; 2 Trauma Nursing Research Center, Kashan University of Medical Sciences, Kashan, Iran; 3 Department of Epidemiology & Biostatistics, Kashan University of Medical Sciences, Kashan, Iran

**Keywords:** Mentorship, Physical examinations, Education, Multimedia, Nursing

## Abstract

**Background:**

Poor competence in clinical examination skills among nurses has been reported in practice, and there is a strong consensus that physical examination (PE) education must be improved. However, deficiencies cannot be resolved by theoretical training alone, and new training approaches are required to enable nursing students to perform PE accurately. This study aimed to determine and compare the effect of two new educational methods (mentorship and educational video methods) on the physical examination skills of Iranian nursing students.

**Methods:**

This study was a clinical audit with three group pretest posttest design. Eligible nursing students were recruited through the census method and assigned to three groups (mentorship, educational videos, and control group) using permuted block randomization. Students were taught PE skills in three vital systems through three approaches (mentors, educational videos, and routine educational methods). Data were collected using a two-part instrument consisting of a demographic information questionnaire and a 32-item checklist for assessing the students’ skills in examining the respiratory system (10 items), cardiovascular system (13 items), and 12 cranial nerves (9 items). Data were analyzed using SPSS software version 16 and descriptive and analytical statistical tests.

**Results:**

At baseline, students in all groups scored less than half of the possible scores in all three systems, and the mean scores of the three groups were not statistically different (*P* > 0.05). After the intervention, the mean scores of students in the mentorship group increased significantly in all three systems (*P* < 0.001), whereas the mean scores of students in the educational video group and the control group did not change significantly (*P* > 0.05). Furthermore, after the intervention, the mean scores of the educational video group and the control group did not significantly differ in any of the three systems (*P* > 0.05). The ANCOVA showed that with posttest score as the covariate, PE skills in all three systems improved one week after the intervention in the mentor group compared to the control group and the educational video group. However, PE skills in all three systems did not improve one week after the intervention in the educational video group compared to the control group.

**Conclusions:**

The mentorship method is more effective than the educational video and routine methods for teaching PE skills to nursing students. Nursing schools can use the mentor method in training nursing students, and it is recommended to revise the PE lesson unit in the nursing curriculum and exchange it from a purely theoretical-based unit to a mixture of theoretical and clinical-based training. Educational videos alone cannot improve nursing students’ PE skills; thus, if educational videos are used to teach clinical skills, it is necessary to hold hands-on sessions to provide feedback to students and correct their mistakes.

**Supplementary Information:**

The online version contains supplementary material available at 10.1186/s12912-023-01626-w.

## Background

Health assessment is a crucial responsibility of nurses and serves as the initial step in the nursing process [1]. Physical examination (PE) is one of the most important components of health assessment, and nurses should be competent in this are Physical examination (PE) is a critical component of health assessment, and it is essential for nurses to possess the necessary skills in this area [2]. A thorough physical examination aids in gathering and analyzing vital information about the patient’s health status, establishing effective communication with patients, diagnosing patient problems, and enhancing the relationship between nurses and physicians [1]. Nurses’ proficiency in PE enables them to identify patient issues, promote patient safety, and improve the quality of care provided [2, 3].

Literature reviews have indicated that nurses and nursing students receive inadequate training in PE skills, and they do not conduct patient health assessments correctly or comprehensively in clinical settings [4–6]. The Iranian nursing curriculum includes a one-unit lesson on PE, consisting of nine 90-minute sessions. The course is primarily theoretical, with some practical sessions conducted in a skill lab center using simulators. However, there is no clinical training for PE skills. This lack of knowledge and poor clinical competence in examination skills is not unique to Iran and has been reported in other countries. Research has shown that nurses utilize less than half of what they learned in physical assessment courses at universities [7], and their use of PE skills in patient care is limited to checking vital signs [2, 8–10]. It is widely acknowledged that PE education must be improved, but theoretical training alone cannot address these shortcomings. Therefore, novel training approaches are required to enable nursing students to perform PE accurately.

Moreover, the limited capacity of nursing education in clinical practice poses a significant challenge to the nursing profession, and it is crucial to ensure that it meets the needs of these professionals. To address these issues, researchers have attempted various training approaches, including simulation debriefing, hybrid learning, problem-based learning, multimedia teaching methods, preceptors, mentors, and technology-optimized practice processes [11–15], to enable nursing students to perform accurately in clinical settings. However, there is limited literature on the best practices for teaching PE [16].

Mentorship is one of the most effective methods for teaching clinical skills. Studies have shown that this approach increases interest in the profession, enhances self-confidence, reduces stress and anxiety [17–20], and improves mentees’ knowledge and skills [21, 22]. However, some studies have reported that the mentorship did not significantly affect the academic achievement and skills of nursing and medical students [23, 24]. In this method, an experienced person (the mentor) tries to guide, support, and trains a mentee and facilitates the mentee’s personal and professional growth and development by promoting an effective relationship [25].

Educational videos—as a multimedia teaching method—are also increasingly being used to teach patients and students. By combining text, videos, images, sounds, and animations and allowing repetition, this method applies multiple senses of learners to facilitate learning [26]. In a study of Taiwanese nursing students, educational videos significantly improved students’ knowledge and skills in urinary catheterization [27]. A study also compared video-based education with conventional instruction (including lectures and simultaneous demonstration) and reported that video-based education was more effective in teaching surgical hand-washing to medical students [28]. In contrast, a study also reported that the use of educational videos alone did not improve the skills required for peritoneal dialysis in nursing students [29]. In an effort to find an effective educational method for improving PE skills, researchers decided to assess new educational methods to enable nursing students for better future clinical performance.

### Nursing curriculum in Iran

The undergraduate nursing program in Iran is based on a standardized curriculum offered by the Ministry of Health and Medical Education. The program consists of eight semesters over four years, during which nursing students must pass 136 lesson units. The first three years of the program focus on theoretical learning, with 95 units being theoretical-based and the remaining units (18 units) being clinical-based. In hospitals, students are divided into small groups of 4–5 and taught by a faculty member with direct supervision. During the senior year, students participate in a clinical internship course designed to facilitate the transition from theory to practice and prepare them to work as independent nurses. During this stage, students work under the direct supervision of hospital staff and the indirect supervision of faculty members.

### Theoretical framework

Various adult learning theories have been suggested to understand the complex processes of higher education. These theories look at different aspects of knowledge and skills acquisition; the ultimate task of a learner is to achieve mastery in the chosen field while being a lifelong learner. Mentorship is an adult learning theory that involves a highly empathic experienced person (the mentor) guiding another person (the mentee) in the development and re-analysis of ideas, learning, personal, and professional development. It is a crucial part of graduate education and helps develop training skills. Mentors carry out four leadership behavior styles: directing, coaching, supporting, and delegating. They combine the improvement of technical skills with personal development to ensure the progression of the mentee in their academic field [30, 31].

Based on the mentoring theory, the researchers developed a study design to compare two educational methods with routine educational content for better clinical performance among nursing students.

### Aim

The aim of the study was to determine and compare the effect of mentorship and educational video methods on physical examination skills among Iranian nursing students.

### Research hypotheses

H1: Compared to the control group, the intervention group with mentorship method will improve PE skills scores in nursing students.

H2: Compared to the control group, the intervention group with video-based method will improve PE skills scores in nursing students.

H3: Compared to the video-based group, the mentorship group will improve PE skills scores in nursing students.

## Methods

### Study design and participants

This study was a clinical audit with three group pretest posttest design which conducting in 2021 on 68 undergraduate nursing students at the school of nursing and midwifery of Kashan University of Medical Sciences.

Eligible students were recruited through census method, so that all students in 7th and 8th semesters assigned into three groups by the permuted block randomization method using online software (i.e., www.sealedenvelope.com). The participants were divided into three groups including mentorship, educational video and control with blocks of six.

Inclusion criteria consisting passing the theoretical health assessment course, owning a smartphone or access to audiovisual facilities for the educational video group, no work experience in the clinical setting other than student work. Exclusion criteria included missing one or more educational sessions, and not watching the educational video according to the specified schedule (in the educational video group) and the allowance to withdraw from the study whenever they want.

### Study instruments

A research-made, two-part instrument was used for data collection. The first part was a demographic information questionnaire that included questions about students’ age, gender, student work experience, place of residence, and study semester. The second part of the instrument was a 90-item checklist designed based on the “Bates Guide to Physical Examination and History Taking” textbook [32]. The checklist included 90 items for assessing the students’ skills in examining the patients’ respiratory system (10 categories, 27 items), cardiovascular system (13 categories, 36 items), and 12 cranial nerves (9 categories, 27 items).

Each skill was further divided into smaller components and scored as either Yes = 1 or No = 0. Consequently, the maximum scores for respiratory, cardiovascular, and nervous system skills were 27, 36, and 27 respectively, with the minimum score being zero. The content validity of the checklist was confirmed by 10 expert faculty members from the School of Nursing and Midwifery at Kashan University of Medical Sciences. The reliability of the checklist was assessed using the inter-rater agreement method. To achieve this, two assessors simultaneously completed the checklist for 10 students who had successfully completed the health assessment course but had not yet commenced their internship. Subsequently, the interclass correlation coefficient was calculated as 0.88, 0.84, and 0.81 for the respiratory, cardiovascular, and nervous systems respectively.

The OSCE (Objective Structured Clinical Examination) method was utilized to evaluate students’ skills. It was designed with three separate stations for each of the three systems: one station for the respiratory system, one for the cardiovascular system, and one for the cranial nerves. Three experts, who were faculty members at Kashan University of Medical Sciences, evaluated the students’ physical examination (PE) skills using the OSCE method. Each expert tested one system using a checklist, meaning that one faculty member examined the respiratory PE skills of all participants, the second examined the cardiovascular PE skills, and the last examined the cranial nerve PE skills. The same experts were used for each system and at two different time points: before and after the intervention. The experts were unaware of which students belonged to which group.

At the beginning of the study, a pretest was taken from all participants using a researcher-made checklist. The students were then allocated into three groups (mentorship, educational video, and control) in blocks of six based on permuted block randomization.

### Preparation and intervention

#### Mentor group

To select a mentor, a call was made at the university to find the best candidate among volunteer students in the master’s degree program. Five students in the field of medical-surgical nursing were selected. These students underwent five two-hour sessions where they were taught about the principles of mentoring, the characteristics of a good mentor, and how to teach PE skills to nursing students in a clinical setting. Their technical competencies for the mentor role were tested and confirmed by a nursing professor, and two students with high scores were chosen as mentors.

After the pretest and allocation to the mentor group, a three-day mentorship program was planned over three consecutive weeks, with each system being taught on a different day. The mentor performed physical examinations on real patients in a real clinical setting, with informed consent obtained from each patient. The mentor allocated 45, 60, and 35 min for teaching the PE skills of the respiratory system, cardiovascular system, and nervous system, respectively. Afterward, the students were asked to examine the patient again in the presence of the mentor, who would correct any mistakes. Although each session with the mentor in the clinical setting was approximately 3 h, there was flexibility in the timing. Finally, a post-test was administered one month after the intervention (one week after the mentorship program).

### Educational video group

Firstly, some educational videos were collected from valid sources (www.batesvisualguide.com website) and then were approved by three faculty members who were experts in the field of health assessment. Then three educational videos were selected in which every system was taught separately. Times for respiratory system, cardiovascular system and nervous system were 45, 60 and 35 min.

After pretest and allocating to educational video group, students received educational videos on cardiovascular, respiratory, and nervous system examination weekly via Telegram messenger. Totally, three educational videos were sent to each student in three consecutive weeks. Students were supposed to learn PE skills with videos as a self-study. Because the mentor group stay in clinical setting with mentor and exercise what they learn, the research team decided to ask students to watch each educational videos two times in the desired week. Finally, a post-test was taken after a month (one week after intervention).

### Control group

After pretest and allocating to control group, they did not receive any special training. They just received routine educational contents based on nursing curriculum.

As participants in the clinical internship stage, these students went to the hospital and received clinical training under the direct supervision of staff and indirect supervision of a faculty member. The educational content for the control group consisted of three one-hour sessions, with each session focusing on one system. These sessions were delivered through lectures and were also approved by three faculty members who were experts in health assessment. Students were instructed to study the content and perform physical examinations on patients based on what they had learned at the hospital. The timing of the study was determined by the pretest and post-test, which were conducted at the beginning and end of the study period. At the conclusion of the study, the educational videos’ content was provided to the control group to ensure educational justice and equity among all participants.

### Ethical considerations

This study was approved by the Ethics Committee of the School of Nursing and Midwifery, Kashan University of Medical Sciences (ethics code: IR.KAUMS.NUHEPM.REC.1399.057). The objectives of the study were explained to all participants and informed consent was obtained from all participants. The students were also assured that their personal information would be kept confidential, they could withdraw from the study at any time, the study would not harm them, and the scores obtained in the study would not affect their internship grades. In addition, all patients who were examined in the mentorship group signed a written informed consent form and were assured that the study would not harm them and that they were free to participate in the study.

All rights of the participants were respected in accordance with the Helsinki Declaration.

### Data collection

All graduating nursing students who met the inclusion criteria were invited to participate in the study. Data collection was carried out with a reliable and valid research made checklist for assessing PE skills. For comparing the effect of intervention, the checklist is completed in two time points. One before an intervention (pretest) and other one-month after intervention (posttest).

### Data analysis

The SPSS software version 16 was used for data analysis. Normality was tested by the Kolmogorov–Smirnov test, and descriptive statistics (frequency, percentage, mean, and standard deviation) were calculated for the demographic variables. The categorical demographic characteristics of the three groups were compared using the chi-square test, while their mean age and grade point average were compared using the one-way analysis of variance. One-way analysis of variance and Tukey post hoc test were also used to compare the mean scores of the PE skills of the students in the three groups. Furthermore, the paired t-test was used for the within-group comparison of the mean PE skills. The significance level was set at < 0.05.

## Results

22 people in the control group, 21 people in the mentorship group, and 21 people in the educational video group completed the study. One student from the control group, one student from the mentorship group, and two students from the educational video group were excluded from the study because of absence in the posttest. Most students were female (56%), native (54%), and had no history of student work (62%). The two groups were homogeneous in terms of demographic characteristics (Table 1).

**Table 1:** Frequency distribution of the subjects’ demographic variables

Characteristics		Educational video	Mentorship	Control	Test result*
		Frequency (%)	Frequency (%)	Frequency (%)	
Gender	Female	11 (52.4)	13 (61.9)	12 (54.4)	χ^2^=0.427 df = 2 *p* = 0.808
	Male	10 (47.6)	8 (38.1)	10 (45.5)	
Place of residence	Dormitory	11 (52.4)	8 (38.1)	10 (45.5)	χ^2^=0.865 df = 2 *p* = 0.649
	Home	10 (47.6)	13 (61.9)	12 (54.5)	
Student work experience	No	12 (57.1)	12 (57.1)	16 (72.7)	χ^2^=1.496 df = 2 *p* = 0.473
	Yes	9 (42.9)	9 (42.9)	6 (27.3)	
Semester	7	12 (43.8)	7 (33.3)	9 (40.9)	χ^2^=2.529 df = 2 *p* = 0.282
	8	9 (56.3)	14 (66.7)	13 (59.1)	
Age (Mean ± SD)		22.86 ± 1.014	22.67 ± 0.483	22.50 ± 0.802	F = 1.080 *p* = 0.346
Grade point average (Mean ± SD)		15.91 ± 1.2632	16.63 ± 1.324	16.12 ± 1.178	F = 1.861 *p* = 0.164*

* Analysis of variance

At baseline, students in all groups scored less than half of the possible scores in all three systems, and the mean scores of the three groups were not statistically different (*P* > 0.05). After the intervention, the mean scores of students in the mentorship group increased significantly in all three systems (*P* < 0.001), whereas the mean scores of students in the control group and the educational video group did not change significantly (*P* > 0.05). In fact, students in the mentorship group scored higher in all three systems after the intervention. Analysis of variance showed that after the intervention, the between-group difference was statistically significant in all three systems (*P* < 0.05). The Tukey post hoc test also showed that the mean scores of students in the mentorship group were significantly different from those of the other two groups in all three systems (*P* = 0.05), whereas the control group and the educational video group were not significantly different in any of the three systems (*P* > 0.05) (Table 2).

**Table 2:** Comparison of the students’ mean scores for respiratory, cardiovascular, and nervous system examination skills, before and after the intervention trough OSCE method

System	Time	Group			Test Result
		Educational video	Mentorship	Control	
		Mean ± SD	Mean ± SD	Mean ± SD	
Respiratory	Before the intervention	11.95 ± 3.58	11.33 ± 4.12	12.14 ± 4.45	F = 0.277 *p* = 0.798
	After the intervention	12.48 ± 3.73	15.71 ± 5.28	12.45 ± 4.26	F = 3.727 *p* = 0.030
	Results of paired t-test	t = 1.759 *p* = 0.094	t = 9.520 *p* < 0.001	t = 0.979 *p* = 0.339	
Cardiovascular	Before the intervention	11.81 ± 5.12	11.14 ± 3.13	12.32 ± 3.95	F = 0.433 *p* = 0.651
	After the intervention	12.38 ± 4.93	18.48 ± 4.27	12.59 ± 3.85	F = 13.27 *p* < 0.001
	Results of paired t-test	t = 1.922 *p* = 0.069	t = 10.11 *p* < 0.001	t = 1.240 *p* = 0.229	
Nervous	Before the intervention	7.90 ± 3.23	8.38 ± 2.17	8.77 ± 2.13	F = 0.617 *p* = 0.543
	After the intervention	8.00 ± 3.83	12.10 ± 2.70	9.05 ± 2.45	F = 10.25 *p* < 0.001
	Results of paired t-test	t = 0.244 *p* = 0.809	t = 8.011 *p* < 0.001	t = 1.188 *p* = 0.248	

* Analysis of variance

The ANCOVA was used to determine which group comparisons are statistically significantly different. (Table 3) The ANCOVA showed that with posttest score as the covariate, PE skills in three systems improved one week after the intervention in the mentor group compared to the control group and the mentor group compared to the educational video group. However, PE skills in three systems were not improved one week after the intervention in the educational video group in comparison with the control group.

**Table 3:** Comparison of the students’ mean scores for respiratory, cardiovascular, and nervous system examination skills between three groups

Dependent variable	Before intervention	After intervention	Mean Difference (I-J)	Std. Error	Sig.^b^	F	Sig.	Partial Eta Squared
respiratory system	control	mentor	-4.075^*^	0.522	0.001	38.038	0.001	0.559
		video	− 0.208	0.521	1			
	mentor	control	4.075^*^	0.522	0.001			
		video	3.866^*^	0.528	0.001			
	video	control	0.208	0.521	1			
		mentor	-3.866^*^	0.528	0.001			
Cardiovascular system	control	mentor	-4.075^*^	0.522	0.000	71.652	0.001	0.705
		video	− 0.208	0.521	1			
	mentor	control	4.075^*^	0.522	0.001			
		video	3.866^*^	0.528	0.001			
	video	control	0.208	0.521	1			
		mentor	-3.866^*^	0.528	0.001			
Nervous system	control	mentor	-3.436^*^	0.527	0.001	29.611	0.001	0.479
		video	0.191	0.531	1			
	mentor	control	3.436^*^	0.527	0.001			
		video	3.626^*^	0.534	0.001			
	video	control	− 0.191	0.531	1			
		mentor	-3.626^*^	0.534	0.000			

ANCOVA

## Discussion

In this study, the effects of mentorship and video-assisted teaching on PE skills of graduating nursing student were compered. The results showed that the mentorship method significantly improved students’ PE scores. However, no significant changes were observed in the students’ mean scores in the control group and in the educational video group. These findings are consistent with Tejos et al. (2021), who found that peer mentoring was as effective as faculty teaching in improving suturing skills [33]. Aslan et al. (2021) also reported that peer mentoring not only improved students’ skills in vital signs monitoring, medication preparation, and subcutaneous injections, but also induced them less clinical stress [34]. Studies have also shown that mentorship training increases students’ enjoyment of learning, self-confidence, self-efficacy, and professional satisfaction [24, 35]. It seems that the mentorship method can effectively improve the PE skills of nursing students. Therefore, it can be recommended that senior undergraduate and graduate students who have received the necessary training for the role of mentor, teach the clinical skills such as PE to nursing students. However, the effectiveness of the mentorship method depends largely on the proper preparation of mentors. If mentors are not adequately prepared for their educational role, the mentorship method may cause students to lose confidence in the educator, reduce their desire to learn, and decrease their professional interest [18].

Contrary to our findings, Lemak et al. (2020) conducted a study to measure suturing skills of nursing students and reported that although the scores of students in groups trained by peer mentors and faculty members were slightly better than those trained by video, the difference was not statistically significant. However, students trained by faculty and peers were more satisfied with their learning because they had the opportunity to receive feedback while demonstrating the skills. Students trained by peer mentors also experienced less anxiety during training sessions [36]. Azadi et al. also compared the effects of faculty-led, clinical mentors, and peer mentoring on nursing students’ wound dressing skills and reported that the group trained by clinical mentors performed best on an OSCE. Although students trained by faculty members and peer mentors scored higher in posttest, their posttest mean scores did not differ significantly [37].

The present study found that the use of educational videos alone did not improve nursing students’ PE skills. Therefore, despite the convenience and low cost of video-assisted education, this method alone may not be effective in enhancing technical skills like PE. However, there are conflicting results regarding the effectiveness of educational videos in teaching clinical skills. For instance, Giroux et al. (2021) reported that video-based training of pelvic examination was as effective as practical training. The researchers attributed the insignificant difference between the two methods to the fact that the pelvic examination is easier compared with other examinations [38]. Another possible reason for the difference between our findings and what Giroux et al. reported could be the time between the end of training and the posttest. In the present study, the posttest was conducted one week after the intervention, whereas Giroux et al. conducted the posttest immediately after the intervention. However, Hošnjak et al. (2019) have shown that video-assisted education is not sufficient for intravenous injection training unless it is supplemented by hands-on training and feedback from an expert [39]. A study by Lee et al. (2019) also showed that the combination of traditional methods and educational videos is more effective in teaching tracheal intubation and airway care skills [40]. Eimer et al. (2020) also noted that the lack of live experience, the absence of feedback, and lack of live interaction between teacher and learner makes educational videos ineffective for teaching psychomotor skills [41]. The presence of an experienced person who provides feedback to learners is an important factor in education. Students are also interested in receiving feedback from an experienced person [40, 42]. It is appeared educational videos alone cannot improve nursing students’ PE skills. Therefore, if educational videos are utilized for teaching clinical skills, it is essential to incorporate hands-on sessions to provide feedback to students and correct any mistakes they may make.

The present study showed that all three groups of students scored less than half of the possible score in the pretest. This finding shows that students did not prepare adequately in the PE course. Jaberi et al. (2019) also reported that nursing students do not have sufficient skills in PE [43]. The lack of sufficient equipment, deeming PE unrelated to nurses, a heavy workload, and lack of time to perform examinations are the most important factors that prevent nurses from performing PE [6, 44]. Therefore, nurse education officials must pay more attention to the training of PE, especially the practical training for these examinations.

### Study limitations

The large number of lesson units during the internship period may have affected the results of this study. It would be better to measure the effect of intervention at three time points: baseline, immediately after the intervention, and, for example, one month later. However, our participants were busy with their internship course and did not accept to participate for more time.

In the mentorship group, small groups of six students were used, and as a result, they benefited from both mentor training and small group training. It was not possible to separate these two effects in this study. Future studies with new designs are needed to separate this effect.

### The implications for research and practice

It is clear that the mentorship approach can effectively improve the PE skills of nursing students. Therefore, it is highly recommended to use mentorship training for the clinical examination unit to nursing students or even other medical sciences students. Furthermore, although educational videos alone cannot improve nursing students’ PE skills, it seems that it is vital to incorporate hands-on sessions to provide feedback to students and correct any mistakes they may make. It is recommended to compare the effect of mentor-mentee training with mentor-mentees (small group of mentees) training at three different time points.

## Conclusions

The present study showed that the mentorship method is more effective than educational video and the routine method for teaching PE skills to nursing students. Therefore, two research hypotheses (H1and H3) are accepted in this study. Accordingly, nursing schools can use mentor method in training of nursing students. It is recommended to revise this lesson unit (PE) in nursing curriculum and exchange it from purely theoretical based unit to a mixture of theoretical- clinical based training. In addition, It also appears educational videos alone cannot improve nursing students’ PE skills. Thus, if educational videos are used to teach clinical skills, it is necessary to hold hands-on sessions to provide feedback to students and correct their mistakes.

### Electronic supplementary material

Below is the link to the electronic supplementary material.

Supplementary Material 1

## Data Availability

The datasets used and analyzed during the current study are available from the corresponding author on reasonable request.

## Abbreviations

PE: Physical Examination
OSCE: Objective Structured Clinical Examination
χ2: Chi-square test
df: Degrees of freedom
M ± SD: Mean ± Standard Deviation
