# Supplementary material for: The effects of mentorship and educational videos on nursing students’ physical examination skills: a clinical audit
Source: BMC Nurs. 2023 Dec 6;22:463. doi: 10.1186/s12912-023-01626-w (PMC10698891; doi:10.1186/s12912-023-01626-w)
Supplement: Supplementary file 1 — Supplementary Material 1 [file 12912_2023_1626_MOESM1_ESM.docx]

**Physical examination checklist**

Gender: Female male

Age: ……..

Grade point average: ……..

Place of residence: Dormitory Home

Student work experience: yes No

Semester: term 7 term 8

| **The Respiratory Assessment** | | | |  |
| --- | --- | --- | --- | --- |
| **Chest inspection** | **Steps** | **Yes** | **No** |  |
|  | Communicate with the patient and explain the reason for doing the work. |  |  |  |
|  | Stand behind the head or in front of the patient |  |  |  |
|  | Declare any deformation or asymmetry in the chest. |  |  |  |
|  | Report any abnormal indentation of the intercostal spaces in the inspiration. |  |  |  |
| **Chest palpation** | | | |  |
|  |  | **Steps** | **yes** | **No** |
|  | **lung Expansion** | Stand behind the patient |  |  |
|  |  | Put the thumbs approximately at the level of the 10th rib, so that the other fingers are parallel to the outer part of the rib cage and embrace it. |  |  |
|  |  | At the same time, put hands in this area and slide them inwards |  |  |
|  |  | ask the patient to take a deep breath |  |  |
|  |  | **Steps** | **yes** | **No** |
| **Tactile fremitus** | Use the bony part of the palm at the base of the fingers to the middle and side |  |  |  |
|  | Place your hand on the patient's back between the shoulders |  |  |  |
|  | Ask the patient to announce the number 44 or a similar word |  |  |  |
| **Chest percussion** | | | |  |
|  | **Steps** | **yes** | **No** |  |
| **Preparation phase** | Stand on the patient's side or back. |  |  |  |
|  | Put the middle finger of the left hand in a fully straightened position and firmly press the distal joint on the surface to be tapped. |  |  |  |
|  | Avoid contact of any other part of the hand with the target surface (even fingers) |  |  |  |
|  | He strikes with quick and sudden but loose wrist movements |  |  |  |
|  | Blows hit the distal intervertebral joint. |  |  |  |
|  |  | **Steps** | **yes** | **No** |
|  | **Diaphragm identification** | It is placed on the back of the patient |  |  |
|  |  | Put the finger of the pleximeter above the middle part of the breast and the outer area and tap gradually downwards. |  |  |
|  |  | When the dull sound is replaces with the resonance sound clearly, it declares it as the location of the diaphragm |  |  |
|  | **Pulmonary Auscultation** | | | |
|  | **Steps** | **yes** | **No** |  |
| **Preparation phase** | Before auscultation, ask the patient to cough 1-2 times to clear the extra sounds. |  |  |  |
|  | Ask the patient to breathe deeply with the open mouth. |  |  |  |
|  | Put the diaphragm part of the stethoscope directly on the patient's skin. |  |  |  |
|  | Auscultate both lungs using a ladder pattern |  |  |  |
|  |  | **Steps** | **yes** | **No** |
|  | **Bronchial sound** | To hear this sound, put the stethoscope on the manubrium |  |  |
|  |  | **Steps** | **yes** | **No** |
|  | **Vesicular sound** | To hear this sound, put the stethoscope on the last two thirds of the lung |  |  |
|  |  | **Steps** | **yes** | **No** |
| **Tracheal sound** | To hear this sound, put the stethoscope on the trachea |  |  |  |
|  |  | **steps** | **yes** | **No** |
| **Broncho vesicular sound** | To hear this sound, put the stethoscope in the space between the first and second ribs | **yes** | **No** |  |

|  | **The cardiovascular system** |  |  |  |
| --- | --- | --- | --- | --- |
|  | **Steps** | **Yes** | **No** |  |
| **Blood pressure measurement** | Communicate with the patient and explain the reason for doing the work. |  |  |  |
|  | Ask the patient to rest quietly for 5 minutes. |  |  |  |
|  | Choose a cuff of the right size (two thirds of the arm). |  |  |  |
|  | Put the patient's hand at the level of the heart or on the table |  |  |  |
|  | Put the center of the inflatable part of the cuff on the right side over the brachial artery. |  |  |  |
|  | Put the diaphragm part of the stethoscope at the bottom of the cuff (not below it) and at the location of the brachial artery. |  |  |  |
|  | Start to inflate the cuff and then slowly deflate it |  |  |  |
|  | When the air is empty, call the first sound of two consecutive beats as systole and the place where the beats disappear as diastole. |  |  |  |
|  |  | **Steps** | Yes | No |
| **Intermittent pulse** | Declare the regularity of heart rhythm, weak, strong and alternating arterial pulse pressure during blood pressure control |  |  |  |
|  |  | **Steps** | Yes | No |
|  |  | Inflate the cuff quickly to a pressure higher than the systolic pressure and then deflate it slowly. |  |  |
|  | Declare excessive drop in systolic pressure (more than 10-12 mm Hg) during expiration. |  |  |  |
|  | **Steps** | Yes | No |  |
| **Assessment of jugular vein pressure** | Put a pillow under the patient's head and raise the head of the bed by 30 degrees. |  |  |  |
|  | Turns the patient's head slightly against the direction of examination. |  |  |  |
|  | Find the highest point of the pulse in the jugular vein on the side of examination. |  |  |  |
|  | Put a ruler horizontally (parallel to the highest point of the pulse) and a ruler on the sternal angle vertically. |  |  |  |
|  | Read the height determined on the vertical ruler in centimeters and add 5 cm to it to determine the jugular vein pressure number. |  |  |  |
|  | **Steps** | Yes | No |  |
| **Measurement of central venous pressure** | Put the patient in a supine position. |  |  |  |
|  | Ensure that the central venous line path is open (by washing the line). |  |  |  |
|  | Align the zero mark of the ruler with the phlebostatic axis |  |  |  |
|  | Clamp the place where the liquid enters the patient and at the same time fill the manometer with serum up to 20 cm. |  |  |  |
|  | Open the connection between the patient and the manometer and announce the corresponding number. |  |  |  |
|  | **Steps** | **Yes** | **No** |  |
| **Carotid pulse palpitation** | Raise the head of the patient's bed 30 degrees. |  |  |  |
|  | Place your index and middle finger or thumb in the lower third of the neck on the carotid artery and apply pressure to the back and determine the location of the carotid pulse. |  |  |  |
|  |  | **Steps** | **Yes** | **No** |
|  | **Tremor and bruit of carotid artery** | Ask the patient not to breathe for 15 seconds. |  |  |
|  |  | Place the diaphragm of the phone near the upper end of the thyroid cartilage below the angle of the jaw |  |  |
|  |  | Perform auscultation using the diaphragm of the phone on the carotid pulse. |  |  |
|  |  | **Steps** | **Yes** | **No** |
|  | **Brachial artery pulse** | Use your middle and index finger to control the pulse. |  |  |
|  |  | Place the patient's arm in a position where the elbow is in extension and the palm is up |  |  |
|  |  | Touch the pulse on the inner side of the biceps tendon. |  |  |
|  | **Heart palpitation** | | | |
|  |  | **Steps** | **Yes** | **No** |
|  | **Palpitation of S1 and S2** | Put the patient in a supine position. |  |  |
|  |  | Place the right hand firmly on the chest, touch the carotid artery in the lower third of the neck with the index and middle fingers of the left hand. |  |  |
|  | **Palpitation of apex area** | Determine the location of the apical beat based on the intercostal space (4 or 5) and the distance from the midline of the sternum. |  |  |
|  |  | Put your fingertips in the third, fourth and fifth intercostal space. |  |  |
|  |  | Put your fingertips in the second left intercostal space |  |  |
|  | **Palpation of the lung area** | Tell the patient to hold exhalation and at the same time touch the mentioned space. |  |  |
|  | **Palpation of the aorta area** | Place your fingers in the second right intercostal space. |  |  |

|  | **The nervous system( Cranial nerves)** |  |  |
| --- | --- | --- | --- |
|  | **Steps** | **Yes** | **No** |
| **Olfactory nerve** | Make sure that the nostrils are open by applying pressure on one of the holes and try to breathe through the other hole. |  |  |
|  | Ask the patient to close eyes |  |  |
|  | Test the sense of smell with a familiar smell and do this for the other side as well. |  |  |
|  | **Steps** | **Yes** | **No** |
| **Optic nerve**  **Oculomotor nerve(2 and 3)** | Examine the visual fundus with an ophthalmoscope. |  |  |
|  | Test the field of vision by standing in front of the patient. |  |  |
|  | Check the response of the pupil to close objects. |  |  |
|  | Check the size and shape of the pupils and then compare each side with the other side. |  |  |
|  | **Steps** | **Yes** | **No** |
| **Oculomotor nerve** **Trochlear nerve** **Abducens nerve** | Examine extraocular movements in 6 main directions. |  |  |
|  | Check eye alignment by shaking an object. |  |  |
|  | **Steps** | **Yes** | **No** |
| **Trigeminal nerve** | Ask the patient to press her teeth together while touching the temporal muscle and the masseter muscle in turn - ask the patient to open her mouth and turn the jaw to the sides |  |  |
|  | Before starting, ask the patient to close her eyes, then check the patient's forehead, cheek and jaw on each side for pain with a sharp object - |  |  |
|  | Touch the cornea gently with a little cotton from the corner of the patient's eye without touching the eyelashes. |  |  |
|  | **Steps** | **Yes** | **No** |
| **Facial nerve** | Ask the patient to speak and then declare any asymmetry in the face |  |  |
|  | Ask the patient to raise eyebrows - frown - close both eyes - show her upper and lower teeth - smile - inflate both cheeks. |  |  |
|  | **Steps** | **Yes** | **No** |
| **Auditory/vestibular nerve** | Ask the patient to hold one ear and repeat the whispered numbers in the other ear. |  |  |
|  | Rinne test: Place the diapason 2 inches away from the ear canal hole and in front of the mastoid bone. |  |  |
|  | Declare the normal state if the sound heard from the air conduction is longer than the bone conduction. |  |  |
|  | Weber's test: Place the vibrating diapason on the patient's forehead |  |  |
|  | It is declared normal when the same sound is heard in both ears |  |  |
|  | **Steps** | **Yes** | **No** |
| **Glossopharyngeal nerve**  **Vagus nerve** | Declare the patient's voice whether it is hoarse or nasal. |  |  |
|  | Ask the patient to open her mouth and declare asymmetry in the movements of the soft palate, pharynx, and small tongue |  |  |
|  | Check the patient's gag reflex with a swab. |  |  |
|  | **Steps** | **Yes** | **No** |
| **Accessory nerve** | Place on the patient's back and declare atrophy or fasciculation of the trapezius muscles |  |  |
|  | Ask the patient to raise her shoulder against the resistance of the examiner's hands and declare the strength and contraction of the trapezius muscles. |  |  |
|  | Ask the patient to turn her head to any side against the resistance of the examiner's hands, and declare the contraction of the sternum-clavicular-mammary muscle |  |  |
|  | **Steps** | **Yes** | **No** |
| **Hypoglossal nerve** | declare that the pronunciation of words by the patient is normal |  |  |
|  | Ask the patient to open her mouth and move her tongue and check the atrophy and symmetry of the tongue in the mouth. |  |  |
